# Supplementary material for: Performance evaluation of the Roche Elecsys® Anti-SARS-CoV-2 immunoassays by comparison with neutralizing antibodies and clinical assessment
Source: PLoS One. 2022 Sep 15;17(9):e0274181. doi: 10.1371/journal.pone.0274181 (PMC9477342; doi:10.1371/journal.pone.0274181)
Supplement: Attachment — Submitted filename: Response to Reviewers.docx [file pone.0274181.s001.docx]

March 23, 2022

Ms. Agatha Macaraig

Straive Editorial Assistant

RE: Resubmission of the revised manuscript titled “Performance evaluation of the Roche Elecsys Anti-SARS-CoV-2 immunoassays by comparison with neutralizing antibodies and clinical assessment” by Takei S., et al.

Ms. Macaraig,

We answer the requirements of the journal requirements as follows;

Reply (Answer; A) to the comments and questions (Comment; C):

Journal Requirements:

When submitting your revision, we need you to address these additional requirements.

C1. Please ensure that your manuscript meets PLOS ONE's style requirements, including those for file naming. The PLOS ONE style templates can be found at

https://journals.plos.org/plosone/s/file?id=wjVg/PLOSOne_formatting_sample_main_body.pdf and

<https://journals.plos.org/plosone/s/file?id=ba62/PLOSOne_formatting_sample_title_authors_affiliations.pdf>

**A1. We corrected the manuscript following PLOS ONE's style requirements, including those for file naming.**

C2. We note that the grant information you provided in the ‘Funding Information’ and ‘Financial Disclosure’ sections do not match.

When you resubmit, please ensure that you provide the correct grant numbers for the awards you received for your study in the ‘Funding Information’ section.

**A2. We stated the financial disclosure in the cover letter.**

C3. Thank you for stating the following in the Competing Interests section:

"The reagents used in this study were provided by Roche, but the study was performed by scientifically proper methods without any bias. "

Please confirm that this does not alter your adherence to all PLOS ONE policies on sharing data and materials, by including the following statement: "This does not alter our adherence to PLOS ONE policies on sharing data and materials.” (as detailed online in our guide for authors http://journals.plos.org/plosone/s/competing-interests). If there are restrictions on sharing of data and/or materials, please state these. Please note that we cannot proceed with consideration of your article until this information has been declared.

Please include your updated Competing Interests statement in your cover letter; we will change the online submission form on your behalf.

**A3. We included our updated Competing Interests statement in the cover letter as follows;**

**"The reagents used in this study were provided by Roche, but the study was performed by scientifically proper methods without any bias. This does not alter our adherence to PLOS ONE policies on sharing data and materials.”**

C4. In your Data Availability statement, you have not specified where the minimal data set underlying the results described in your manuscript can be found. PLOS defines a study's minimal data set as the underlying data used to reach the conclusions drawn in the manuscript and any additional data required to replicate the reported study findings in their entirety. All PLOS journals require that the minimal data set be made fully available. For more information about our data policy, please see http://journals.plos.org/plosone/s/data-availability.

"Upon re-submitting your revised manuscript, please upload your study’s minimal underlying data set as either Supporting Information files or to a stable, public repository and include the relevant URLs, DOIs, or accession numbers within your revised cover letter. For a list of acceptable repositories, please see http://journals.plos.org/plosone/s/data-availability#loc-recommended-repositories. Any potentially identifying patient information must be fully anonymized.

Important: If there are ethical or legal restrictions to sharing your data publicly, please explain these restrictions in detail. Please see our guidelines for more information on what we consider unacceptable restrictions to publicly sharing data: http://journals.plos.org/plosone/s/data-availability#loc-unacceptable-data-access-restrictions. Note that it is not acceptable for the authors to be the sole named individuals responsible for ensuring data access.

We will update your Data Availability statement to reflect the information you provide in your cover letter.

**A4. We corrected the Data Availability statement as follows; “All** **relevant data are within the paper.“**

Sincerely,

Yoko Tabe, M.D., Ph.D.

Professor

Department of Clinical Laboratory Medicine,

Juntendo University, School of Medicine
