# Supplementary material for: Performance evaluation of the Roche Elecsys® Anti-SARS-CoV-2 immunoassays by comparison with neutralizing antibodies and clinical assessment
Source: PLoS One. 2022 Sep 15;17(9):e0274181. doi: 10.1371/journal.pone.0274181 (PMC9477342; doi:10.1371/journal.pone.0274181)
Supplement: Attachment — Submitted filename: Response to Reviewers.docx [file pone.0274181.s002.docx]

Reply (Answer; A) to Reviewer’s comments and questions (Comment; C):

**Reviewer 1**

**COMMENTS by Reviewer #1:**

Takei et al. investigated the correlation between the neutralizing antibody titer measured by two commercially available immunoassays and a standard virus neutralization assay. The measurement values of Elecsys Anti-SARS-CoV-2 S and neutralization assays were highly correlated and significantly higher values has been measured in the group of severe and critically ill cases. The study is of interest as serological tests are essential tools to evaluate the neutralizing antibodies targeting the receptor binding domain of the SARS-CoV-2 spike protein.

**COMMENTS (continued):**

The authors might consider some minor comments:
**C1:** Section Materials and methods, Neutralization assay:
It would be interesting to read which strain of SARS-CoV-2 was used in the assay, as the neutralizing activity of the antibodies may be different against the original SARS-CoV-2 strain and the emerging variants of concern. There may be differences between the neutralizing antibody titers measured by the commercially available immunoassays and the protective immunity, depending on the viral variant.

**A1**: Thank you for the valuable suggestions. We added the sentence in the Materials and Methods section and discussed about the differences between the neutralizing antibody titer and the protective immunity, which might depend on the viral variant (L120-123, L275-282).

**C2:** Is there any information available regarding the SARS-CoV-2 vaccination status of these patients? Or they were all immunologically naïve prior to the acute infection?

**A2:** This study targeted only pre-vaccinated patients, who were immunologically naive prior to infection. We added this information to the Materials and Methods section (L98).

**C3**: In addition to the diagnostic PCR assays, has a variant determination been performed in the study group?

**A3:** Unfortunately, no variant determination has been performed in the study group. We mentioned this as the study limitation and discussed the SARS-CoV-2 strain used in the neutralization assay and the SARS-CoV-2 variant prevalent in Japan during the study period (L275-282).

**Reviewer #2:**

In this study, the authors investigated whether measuring anti-SARS-CoV-2 Spike antibodies can predict neutralizing activities in patients’ sera. A commercial assay (Elecsys® Anti-SARS-CoV-2) was utilized for measuring anti-S and anti-N SARS-CoV-2 antibodies. This was then compared to neutralizing titers utilizing an authentic virus neutralization assay.

**C1:** The article is poorly written. All sections need to be improved. There are missing info in the abstract and methods. The authors did not discuss similar studies nor compared their results to literature in their discussion.

**A1:** Following the reviewer’s suggestion, we added more details in the Abstract and Materials and Method section (L44-55, L91-98). We also discussed and compared our results to previous literatures (L248-253, L263-269).

**C2:** A major concern in the study in the correlation analysis. The authors indicated that 146 serum samples were tested, with a positivity rate of >70% for both binding and neutralization assays. However, the correlation figures (Fig 1A and 1B) included only few data points (n=6). All data points (n=146) need to be plotted along with a properly drawn fitted line, in addition, r, and r2 values need to be both mentioned in order to assess the strength of the correlation.

**A2:** One hundred and forty-six samples were included in the correlation analysis. Although some of the plotted sample dots appear to overlap in the graphs in Fig 1A and 1B, all of 146 samples are included in these figures.

We appreciate the reviewer’s comments about the r^2^ values. We added the r^2^ along with the r values (L51-52, L155-156).

**C3:** The abstract needs to be improved. The numbers and percentages need to be added accordingly with each finding.

**A3:** Following the reviewer’s suggestion, we added the numbers and percentages in the Abstract section (L44-55).

**C4:** Patients sample details needs to be better described in the Methods section. There is no information on how many samples were collected per patient. What is the number of inpatients and outpatients? Also, what is the duration between different time-points?

**A4:** We added the detailed information of sample numbers and duration in the Materials and Methods section (L91-97).

**C5:** Figures and legends are poorly presented. Please fix the following:
**a.**　Figure 1 needs re-plotting after including all the results.
**b.**　In figure 2, better to label the lower panels with other labels (E,F and G), better than referring to them as “the lower panel of panel A, B or C”
**c.**　Figure 3 is not clear at all. It is better to use colors to differentiate between patients.
**d.**　In figure 3, why did they choose patients who were tested more than 3 time points only? Patients with 3 time-points can be included in the analysis especially if there is a good time gap between each time-point.
**e.**　All legends need to be expanded to clearly indicate the type of test, analysis, and sample numbers.

**A5:**

a. One hundred and forty-six samples were included in the correlation analysis. Although some of the plotted sample dots appear to overlap in the graphs in Fig 1A and 1B, all of 146 samples are included in these figures.
b. We labeled the lower panels with D, E, and F in Figure 2.
c. We used colors to differentiate between patients in Figure 3.

**d.** We included patients with 3 time-points in the analysis and corrected the sentence in Table 3 abbreviation (L218) and the Result section as follows: “Chronological changes in the results of the neutralization assay, Elecsys® anti-S assay, and Elecsys® anti-N assay were examined simultaneously in 23 inpatients who were tested in three time points or more.” (L203-205).

**e.** Following the reviewer’s suggestion, we added the type of test, analysis, and sample numbers in legends of Fig 1 (L164-170) and Fig 2 (L192-200).

**C6:** In line 260, it is mentioned: “no significant reduction in antibody levels tested was observed until 69 days after the onset of symptoms”. I believe this was observed in 2 patients only whose samples were available at that time-point. This is not enough to draw conclusions. The limitation of sample size need to be clearly mentioned in the discussion.

**A6** Following the reviewer’s suggestion, we deleted the sentence “no significant reduction in antibody levels tested was observed until 69 days after the onset of symptoms” and mentioned the limitation of this study regarding sample size (L274-275).

**C7:** In the abstract, change "levels of neutral antibodies in COVID-19" to "neutralization antibodies"

**A7:** Corrected (L58).

**C8:** The catalog numbers of the used commercial assays need to be mentioned in the Materials and Methods.

**A8:** We added the catalog numbers of the used commercial assays in the methods section (L102-106).

**C9:** Kindly indicate the type/source of the real virus used in the neutralization assay.

**A9:** Information of SARS-CoV-2 strain used in the neutralization assay was provided in the Materials and Methods section (L120-123).

**C10:** In the methods section, it is better to change “Clinical Backgrounds” to “Clinical characteristics”.

**A10:** Following the reviewer’s suggestion, we changed “Clinical Backgrounds” to “Clinical characteristics”　(L86).

**C11:** Table 1. If age is represented as range, please add this to the label of the row “Age range (average)”

**A11:** Corrected “Age, y (average)” to “Age range (average)” (Table 1).

**C12:** Whenever “Elecsys® Anti-SARS-CoV-2” is mentioned, it is better to add “anti-N”, so it does not confuse the reader. This applies to figures and tables too.

**A12:** Following the reviewer’s suggestion, we corrected “Elecsys® Anti-SARS-CoV-2-S” to “Elecsys ® Anti-SARS-CoV-2-S (Elecsys® anti-S)” and “Elecsys® Anti-SARS-CoV-2” to “Elecsys ® Anti-SARS-CoV-2 (Elecsys® anti-N)” in manuscripts, figures, and tables.

**C13:** Table 1 and 3 have the same title. Kindly change the title of table 3 to “inpatients”

**A13:** Thank you for your kind and detailed review. We corrected “patients” to “inpatients” in the title of Table 3.
